# Histological and immunohistochemical analyses of articular cartilage during onset and progression of pre- and early-stage osteoarthritis in a rodent model

**DOI:** 10.1038/s41598-024-61502-8

**Published:** 2024-05-08

**Authors:** Ikufumi Takahashi, Keisuke Takeda, Tadashi Toyama, Taro Matsuzaki, Hiroshi Kuroki, Masahiro Hoso

**Affiliations:** 1 Section of Rehabilitation, Kanazawa University Hospital, 13-1, Takaramachi, Kanazawa, Ishikawa 920-8641 Japan; 2 Department of Motor Function Analysis, Human Health Sciences, Graduate School of Medicine, Kyoto University, Kyoto, Japan; 3 Division of Biostatistics, Innovative Clinical Research Center, Kanazawa University, Kanazawa, Ishikawa Japan; 4 Division of Health Sciences, Graduate School of Medical Science, Kanazawa University, Kanazawa, Ishikawa Japan

**Keywords:** Cartilage, Pathology, Orthopaedics

## Abstract

Early diagnosis and treatment of pre- and early-stage osteoarthritis (OA) is important. However, the cellular and cartilaginous changes occurring during these stages remain unclear. We investigated the histological and immunohistochemical changes over time between pre- and early-stage OA in a rat model of traumatic injury. Thirty-six male rats were divided into two groups, control and OA groups, based on destabilization of the medial meniscus. Histological and immunohistochemical analyses of articular cartilage were performed on days 1, 3, 7, 10, and 14 postoperatively. Cell density of proteins associated with cartilage degradation increased from postoperative day one. On postoperative day three, histological changes, including chondrocyte death, reduced matrix staining, and superficial fibrillation, were observed. Simultaneously, a compensatory increase in matrix staining was observed. The Osteoarthritis Research Society International score increased from postoperative day seven, indicating thinner cartilage. On postoperative day 10, the positive cell density decreased, whereas histological changes progressed with fissuring and matrix loss. The proteoglycan 4-positive cell density increased on postoperative day seven. These findings will help establish an experimental model and clarify the mechanism of the onset and progression of pre- and early-stage traumatic OA.

## Introduction

Osteoarthritis (OA) is an active and dynamic disease primarily of the articular cartilage involving joint components such as the synovial membrane, subchondral bone, and ligaments^1–3^. In recent years, researchers have focused on the two stages of OA: pre- and early-stage OA^4–6^. Pre-OA pertains to a cellular-level stage without pain, motor function problems, or structural changes observable on arthroscopy or magnetic resonance imaging (MRI)^5,7^. Early OA is defined by the onset of pain, slight joint space narrowing on radiographs, and minor histological changes observed on arthroscopy or MRI^6^. Many studies have investigated the histological changes, pain, and specific molecular changes at these stages^8–11^; no consensus has yet been reached on their outcomes. In particular, the histological and immunohistochemical changes that occur over time remain unclear.

Recent fundamental studies on articular cartilage and OA has made great progress by identifying several proteins involved in cartilage metabolism, including Gremlin-1^12^, hyaluronan-binding protein (involved in hyaluronan depolymerization [HYBID])^13,14^, and proteoglycan 4 (also known as lubricin) (PRG4)^15^. In 2019, Gremlin-1, an antagonist of bone morphogenic proteins, was implicated in the Gremlin-1-NF-kB pathway in 2019. This pathway, activated by excessive mechanical stress, involves the interaction between Gremlin-1-NF-kB, inducing matrix metalloproteinases (MMPs), and a disintegrin and MMP with thrombospondin motifs 5 (ADAMTS5)^10^. HYBID, originally described as a deafness gene of unknown function (KIAA1199) and named cell migration-inducing protein, is a key enzyme in hyaluronan degradation, which is a pivotal process in OA. HYBID is reportedly significant in the onset to progression of early OA^13^. PRG4 functions as a proteoglycan, ensuring lubrication in the superficial layer of articular cartilage^15^, and is essential for cartilage maintenance^16^. Despite their presumed involvement in OA onset and progression, examination of these proteins has been limited to the 2-week post-surgical intervention period^12,14,17^. Consequently, the dynamics of these proteins in patients with pre-and early OA remain unclear.

We focused on two types of histological evidence: pre-OA and early OA. In our previous study using a rat model of OA induced by destabilization of the medial meniscus (DMM), surficial fibrillation equivalent to early OA, was observed 14 days postoperatively^18^. Therefore, this study aimed to clarify the histological and immunohistochemical changes within 14 days postoperatively in presumed pre-and early OA using a rat DMM model.

## Results

### General condition

None of the rats (n = 36, six per group) died of postoperative infections or unexpected events during the experimental period. All the rats completed the designated experimental period for each group, demonstrating safety and well-being without adverse events. Weights of the rats in each group are documented in the Supplemental Information (S1 Table).

### Semi-quantitative and qualitative histological analysis

**Figure 1:**
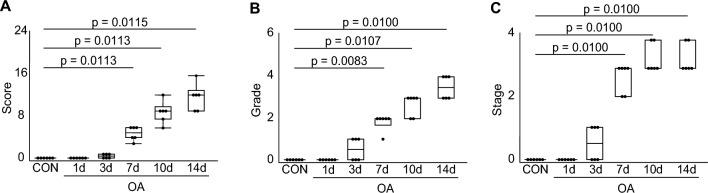
Changes in the Osteoarthritis Research Society International (OARSI) scores over time. (**A**) The OARSI score. (**B**) Grades indicate lesion depth. (**C**) Stage indicating extent of the lesion. The score is the product of the grade and stage. All values began to increase significantly at seven days postoperatively.

The semi-quantitative Osteoarthritis Research Society International (OARSI) evaluation revealed low grades and stages on postoperative days one and three; however, a significant increase was observed after seven days (Fig. 1). The OARSI score, a product of grade and stage values, was also significantly higher in an OA group than that in a control (CON) group at 7, 10, and 14 days postoperatively. The OARSI scores for each group are presented in the Supplemental Information (S2 Table).

**Figure 2:**
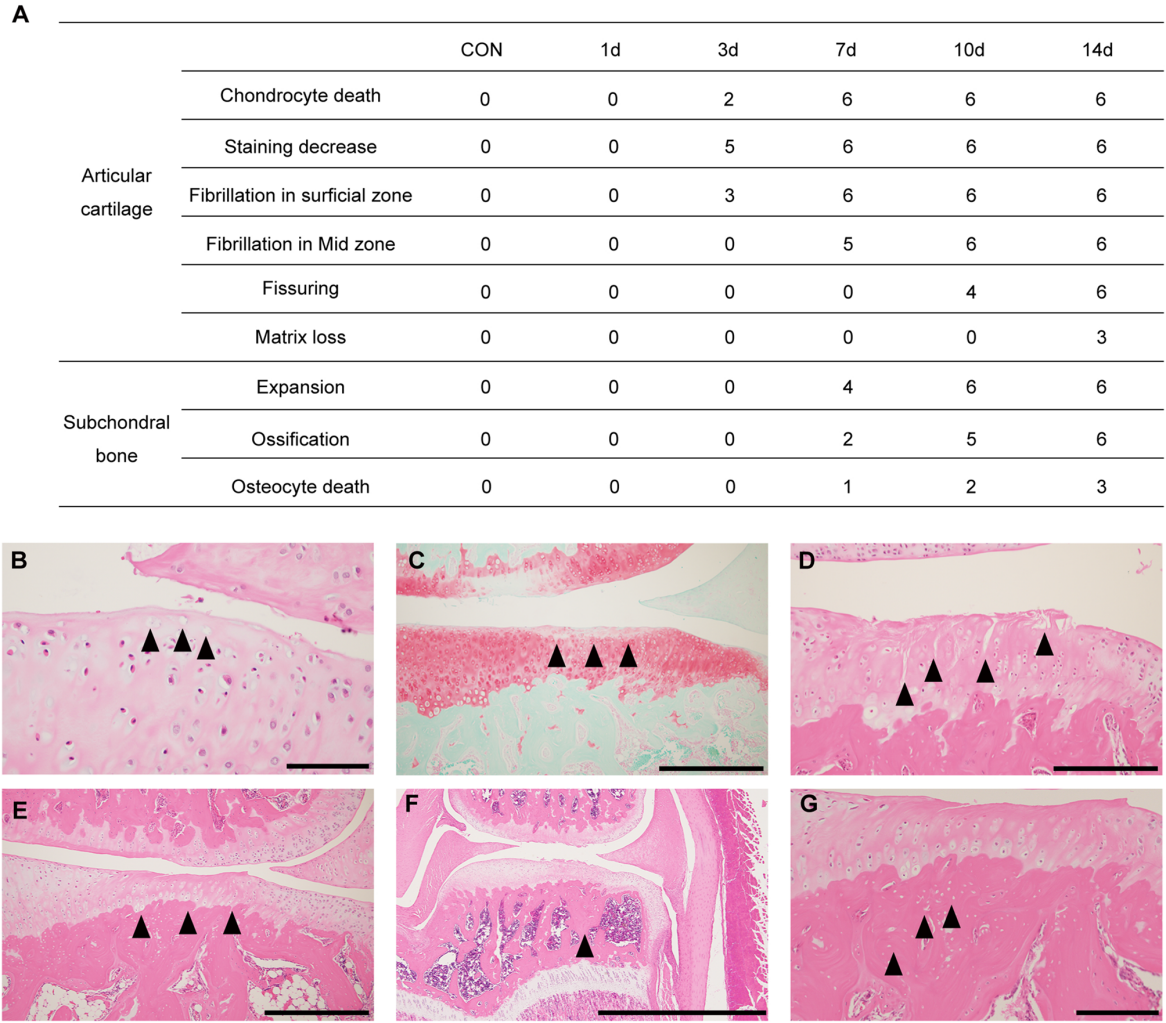
Qualitative assessment of representative histological changes in osteoarthritis (OA). Black triangles indicate the histological changes. (**A**) Number of specimens showing histological changes in articular cartilage and subchondral bone over time. (**B**) Chondrocyte death in the superficial layer of the articular cartilage. (**C**) Decreased matrix staining intensity in the superficial layer of the articular cartilage. (**D**) Fissure reaching deep layers of the articular cartilage. (**E**) Matrix loss accompanied by thinning. (**F**) Ossification of the subchondral bone. (**G**) Osteocyte death in subchondral bone. Scale bar = 100 µm (B), 500 µm (C and E), 200 µm (D and G), and 2 mm (F).

Qualitative histological findings of the articular cartilage are shown in Fig. 2. No representative histological changes indicative of OA was observed in the articular cartilage and subchondral bone in the CON group or on postoperative day one in the OA group. However, three days after the surgery, chondrocyte death, decreased matrix staining, and superficial fibrillation were observed in some specimens. Furthermore, these changes were observed in all the specimens on postoperative days 7, 10, and 14. Fibrillation in the middle layer, fissuring, and matrix loss were observed at postoperative days 7, 10, and 14, respectively. In the subchondral bone, expansion, ossification, and osteocyte death were observed after seven days.

### Histomorphometric analyses

**Figure 3:**
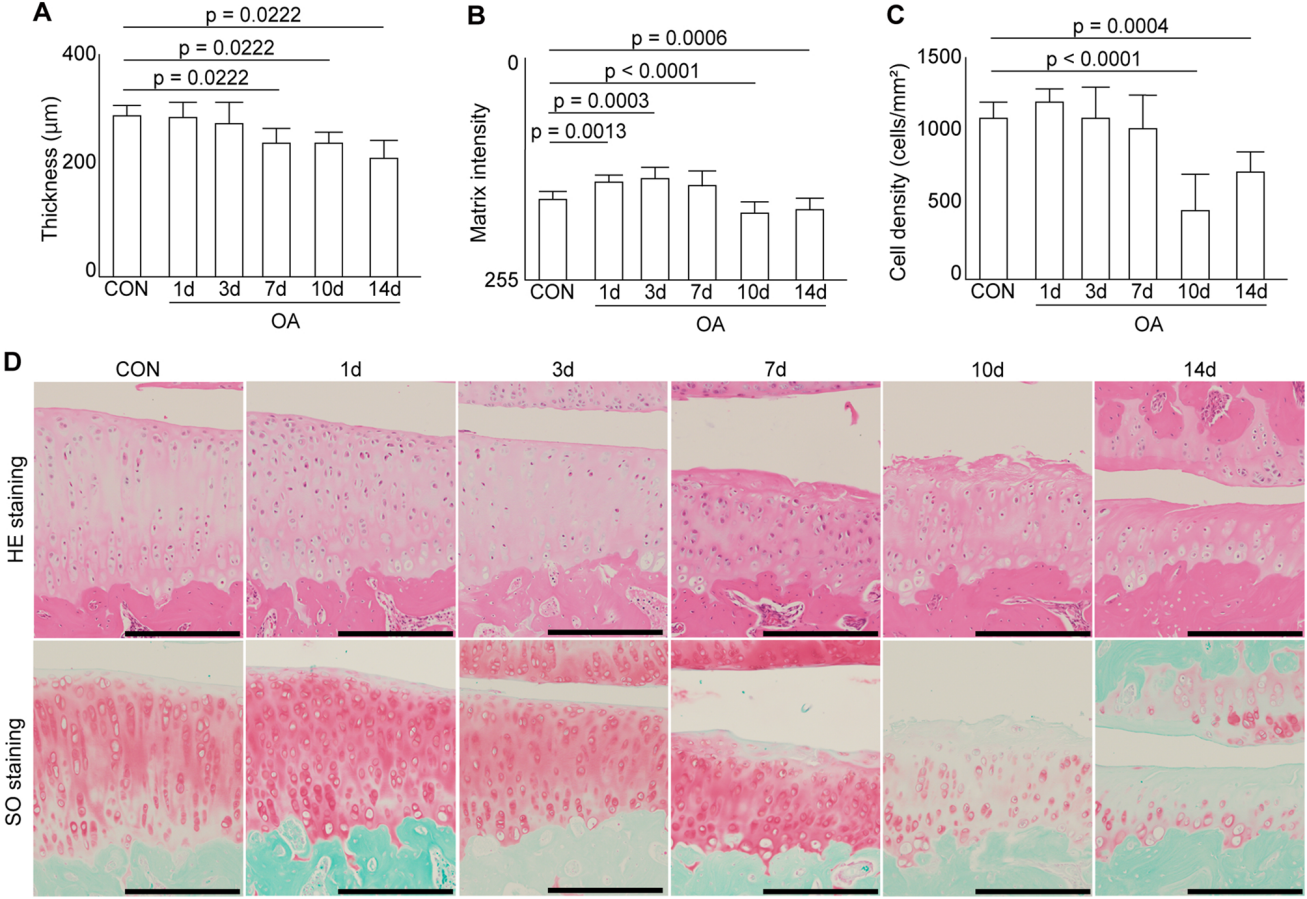
Histomorphological results for the articular cartilage in the medial tibia. (**A**) Articular cartilage thickness. The thickness decreased significantly at seven days postoperatively. (**B**) Matrix intensity stained with safranin O. Matrix intensity increased significantly at one and three days, then decreased significantly at 10 and 14 days. (**C**) Chondrocyte density. Cell density decreased significantly at 10 and 14 days. (**D**) Magnified image of histological changes in the articular cartilage. Scale bar = 200 µm.

No significant differences in cartilage thickness were observed between the two groups at one and three days postoperatively. However, the cartilages of the rats in the OA group were significantly thinner than those of the rats in the CON group after seven days (Fig. 3). Moreover, the intensity of matrix staining with safranin O in the OA group was significantly increased at one and three days postoperatively compared to that in the CON group. After seven days, no significant differences in staining intensity were observed between the two groups. However, after 10 days, the staining intensity in the OA group was significantly lower than that in the CON group. No significant differences in chondrocyte density were observed between the two groups until seven days postoperatively. After 10 days, the cell density in the OA group decreased significantly compared to that in the CON group. The histomorphometric results for each group are presented in Supplemental Information (S2 Table).

### Immunohistochemical analyses

**Figure 4:**
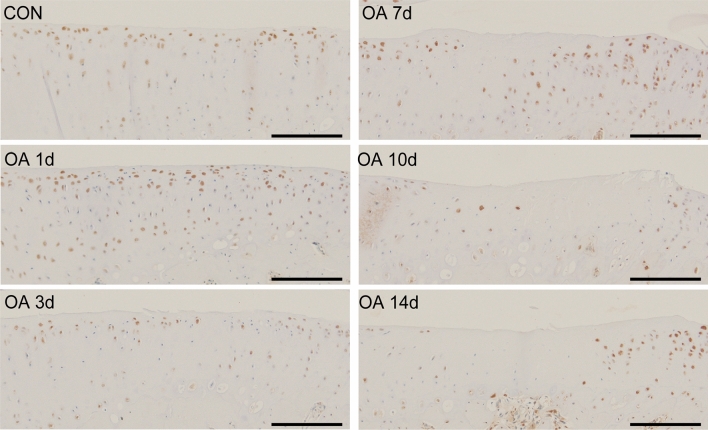
Distribution of cells positive for proteins involved in cartilage degradation in immunohistochemical analysis. Representative immunohistochemical findings showing distribution of HYBID-positive cells. Gremlin-1, MMP13, and ADAMTS5-positive cells were similarly distributed. In the CON group, the positive cells were present from the superficial to middle layers. One day after the surgery, the number of cells increased, and they were present in the superficial to deep layers. After three days, the cells were present just below the lesion. From days 7 to 14, the positive cells shifted to the deeper layers and periphery of the lesion as local histological changes progressed. Scale bar = 200 µm.

**Figure 5:**
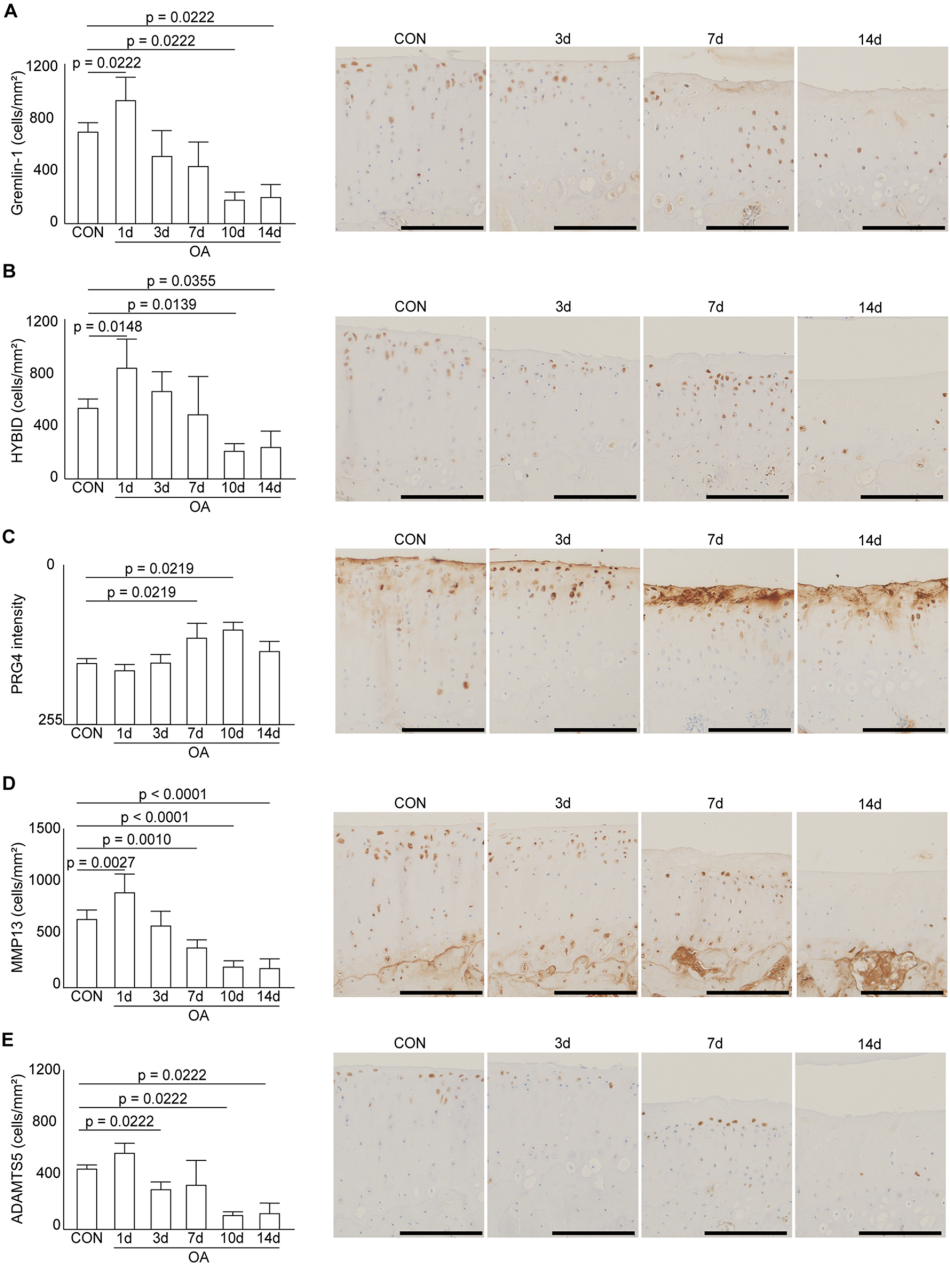
Immunohistochemical results for the articular cartilage in the medial tibia. (**A**) Positive cell density of Gremlin-1. (**B**) Positive cell density of HYBID. (**C**) PRG4 pixel intensity. (**D**) Positive cell density of MMP13. (**E**) Positive cell density of ADAMTS5. Positive cell densities for proteins that degrade cartilages, such as Gremlin-1, HYBID, MMP13, and ADAMTS5, were present at a constant number in the CON group. These proteins and enzymes were significantly increased at one day postoperatively, and then significantly decreased at 10 and 14 days, while PRG4 increased significantly at 7 and 10 days. Scale bar = 200 µm.

In the CON group, all four cell types positive for Gremlin-1, HYBID, MMP13, and ADAMTS5 were observed from the superficial to middle layers (Figs. 4 and 5). On postoperative day one, the number of positive cells increased, and they were distributed in the superficial to deep layers of the cartilage. On day three, the cells were located immediately below the lesion. On day seven, local histological changes progressed (including decreased matrix staining, fibrillation, and fissuring), and more positive cells migrated to the deeper layers and periphery of the lesion. Almost no chondrocytes were observed at the center of the lesion. Instead, it was composed of degenerated cartilage with significantly reduced matrix staining. The areas stained by PRG4 were the superficial chondrocytes and cartilage surfaces in the CON group. However, at 7 and 10 days postoperatively, the staining became more extensive and darker, corresponding to the areas of fibrillation and fissuring.

The positive cell density of Gremlin-1 and HYBID in the OA group significantly increased on postoperative day one compared to that in the CON group (Fig. 5). Density showed no significant difference on days three and seven. However, on days 10 and 14, the density decreased significantly. The PRG4 staining intensity increased gradually over time, with significant differences noted on days 7 and 10. The MMP13-positive cell density in the OA group increased significantly on day one; thereafter, a gradual decrease was observed until it was significantly lower than that of the CON group after seven days. The ADAMTS5-positive cell density increased on day one. After three days, the density of the OA group was significantly lower than that of the CON group. The immunohistochemical results for each group are presented in the Supplemental Information (S3 Table).

## Discussion

**Figure 6:**
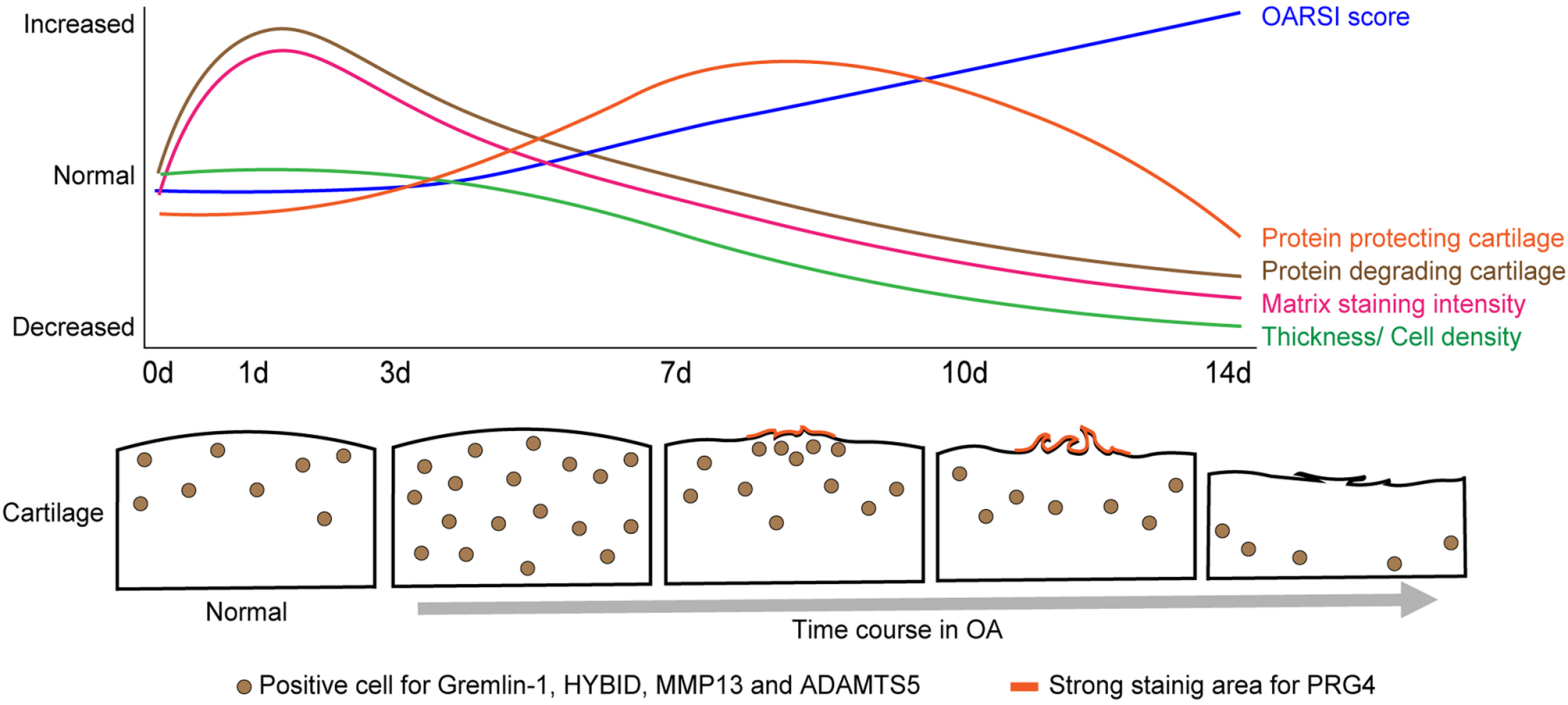
Graphic abstract and possible mechanism of OA onset and progression during the early operative period for the articular cartilage in the post-traumatic model. Histological changes are preceded by an increase in positive cell density for proteins degrading cartilage. The simultaneous increase in matrix staining is presumed to be due to the maintenance of a dynamic metabolic balance. Once the dynamic metabolic balance is disrupted, the histological changes of OA progress with the changing distribution of positive cells for proteins involved in cartilage degradation over time.

We investigated the histological and immunohistochemical mechanisms underlying the early onset and progression of OA using a trauma rat model. Figure 6 shows a graphical abstract, summarizing the novel and informative histological changes and immunohistochemical distributions observed in this study.

In this study, the density of cells positive for Gremlin-1, HYBID, MMP13, and ADAMTS5 proteins involved in cartilage degradation increased on postoperative day one. This preceded the histological changes observed three days postoperatively, including decreased matrix staining and chondrocyte death. Gremlin-1 reportedly activated the Gremlin-1-NFkB pathway under excessive loading, leading to MMP13 expression, which degrades type II collagen^12^. Additionally, NF-kB pathway activation induces ADAMTS5 expression, which degrades aggrecan^19^. HYBID is responsible for hyaluronic acid degradation^10,20^. Therefore, joint destabilization activates cartilage degradation mechanisms early in OA trauma models. Moreover, matrix staining with safranin O increased on postoperative days one and three. This suggests an anabolic response, possibly a compensatory mechanism to counteract the increase in the number of cells positive for cartilage-degrading proteins. The proponents of this study have proposed that increased catabolism triggers homeostasis to maintain the dynamic balance of cartilage metabolism. However, the decline in staining intensity observed on day seven may indicate a breakdown of this compensatory mechanism. After seven days, the cartilage thickness, matrix staining, and cell density rapidly decreased. This was accompanied by an increase in the OARSI scores. The distribution of cells positive for these proteins shifted over time from the superficial layer and center of the lesion to deeper and peripheral areas. This change in the immunohistochemical distribution of positive cells may provide important evidence for the diagnosis and treatment of patients with pre- and early-stage OA.

On postoperative days 7 and 10, PRG4 exhibited increased staining intensity, which was consistent with the localization of histological changes on the cartilage surface, including fibrillation and fissuring. However, PRG4 contributes to joint homeostasis by lubricating the joints and reducing friction on the cartilage surface^16,21^. Previous studies have reported decreased PRG4 expression in drug-induced arthritis and post-traumatic OA models^15,22^. Given the protective effect of PRG4 on articular cartilage, which inhibits cartilage degradation and pain in OA^21,23,24^, the postoperative increase in staining intensity observed in this study likely reflects a compensatory or protective response to surface lesions in the cartilage. However, studies on PRG4 in OA are limited, and the wide variety of OA induction methods, experimental periods, and analytical techniques have led to an incomplete understanding of the role and dynamics of PRG4 in OA. Considering the recent recognition of PRG4 as a possible surface-associated molecule in OA treatment^16^, in future studies, further development and progress on PRG4 are desirable.

In this study, both qualitative and semi-quantitative assessments were conducted to provide a comprehensive histological analysis of the articular cartilage. This approach aimed to objectively capture histological changes through semi-quantitative assessments and sensitively through qualitative assessments. Semi-quantitative analyses, such as the OARSI score, are commonly utilized^25–27^. Qualitative analyses are also important in pathology because they recognize microscopic changes in more detail^28–31^. Qualitative assessment of articular cartilage, biomarkers, and imaging screenings are important to further understand the pathophysiology, diagnosis, and treatment of early OA and pre-OA.

The findings of the present study indicate that the articular cartilage on postoperative day one was characterized by cellular changes without histological alterations. Ryd et al*.* suggested a pre-OA phase in patients with traumatic OA. In this phase, only changes in cellular enzymatic processes were observed, and neither clinical symptoms nor histological changes were present^5,32^. Therefore, the results of this study support the existence of a pre-OA phase. Similarly, minor histological changes in the articular cartilage were observed on postoperative days 3 to 14. Mardy et al*.* proposed an early OA stage based on criteria, such as mild pain, imaging findings, and histological changes^6,7^. Because pain assessment and imaging evaluation were not performed in this study, it was challenging to establish a diagnosis of early-stage OA during the early postoperative period. However, previous studies have not provided detailed histological and immunohistochemical analysis of articular cartilage in the early postoperative period over time^8–11,33–35^. Therefore, the findings of this study regarding the histological changes over time during the early postoperative period, particularly from days 1 to 14, were highly significant. Moreover, previous studies have considered clinical symptoms, imaging findings, and biomarkers as useful candidates for improving the accuracy of pre-OA and early-stage OA^5,7,36^. In the future, histological studies conducted in earlier periods, as seen in this study, can contribute significantly to improving strategies for early diagnosis and treatment of pre-OA and early-stage OA^37^.

The limitations of this study include the absence of a sham model, lack of histological evaluation of other joint components, such as the subchondral bone and synovial membrane, absence of pain and behavioral assessments, and lack of gene expression analyses, such as real-time PCR, on related to the production and degradation of cartilage matrix. Future additions of these analyses to the results of this study will provide a more detailed understanding of the mechanisms underlying the development and progression of OA.

In conclusion, this study revealed the early postoperative histological and immunohistochemical changes in the articular cartilage of a rat model of traumatic OA. This highlights the distribution of Gremlin-1-, HYBID-, and PRG4-positive cells, emphasizing the association between qualitative and quantitative histological changes over time. These findings contribute to the establishment of an experimental model and to the understanding of the mechanisms and progression of pre- and early-stage OA in a rodent trauma model.

## Methods

### Experimental animals and animal care

The study protocol was approved by the Animal Research Committee of the Graduate School of Medicine at the Kanazawa University (Kanazawa, Japan; Approval no. 234389). This study adhered to the ARRIVE guidelines^38,39^ and followed the Guidelines for the Care and Use of Laboratory Animals of Kanazawa University.

Thirty-six 12-week-old male Wistar rats were procured from Japan SLC (Shizuoka, Japan), and a one-week acclimatization period under standard conditions was allowed before commencing the experiments. The rats were caged in pairs in a sanitary, well-ventilated room under controlled temperature and humidity. The rats were subjected to a 12-h light–dark cycle, with ad libitum access to food and water. Health monitoring occurred 2–3 times weekly to assess their general food and water intake, surgical wound condition, and gait. The cages were cleaned by the proponents of the study once or twice every two weeks to sustain a sanitary environment for the rats.

The rats were randomly assigned to two groups (CON [n = 6] and OA induced by DMM [n = 30], with six rats at each time point: 1, 3, 7, 10, and 14 days postoperatively). Throughout the experimental period, the rats in both the CON and OA groups were maintained in a physiological environment and freely ambulated within the confines of their cages. The rats in the CON group, which served as the baseline for normal articular cartilage, were euthanized one week after acclimatization.

### Surgical induction of OA

Destabilization of the medial meniscus surgeries were conducted by the same highly experienced operators (IT and KT), who consistently performed all the procedures^18,40,41^. Induction of DMM involves transection of the medial meniscotibial ligament in the left knee joint^18,41^.

### Preparation and staining of histological specimens

Histological specimen preparation and staining were performed according to the recommendations of previous studies^42,43^ and prior investigations by the proponents of this study^44–46^. Decalcified paraffin sections were prepared for histological examination. Frontal excision of the left knee was performed to assess the histological changes in the medial tibiofemoral joints. The specimens were serially sliced to determine the region in which the articular cartilage of the femur and tibia came into direct contact, while excluding the meniscus. Paraffin sections, measuring 3 µm in thickness, were stained with hematoxylin and eosin and safranin O (0.1%, 5 min). Immunohistochemical staining was performed as described in subsequent subsections. The sections were viewed under a light microscope and documented using a digital camera (BX51 and DP74; Olympus Corporation, Tokyo, Japan) to evaluate histological changes in the articular cartilage. All histological, histomorphometric, and immunohistochemical analyses were performed in a blinded manner.

### Semi-quantitative and qualitative histological analyses for articular cartilage

The OA cartilage histopathology assessment system was used to conduct a semi-quantitative assessment of histological changes in the articular cartilage of the tibia in the medial tibiofemoral joint^47^. The histological scores were determined by a single trained, blinded, independent observer (IT). In a previous study, involving an IT and pathologist (MH), excellent interclass correlation coefficients were obtained for the intra- and inter-rater reliabilities of an OARSI score with 95% confidence intervals of 0.94 (0.92–0.95) and 0.91 (0.89–0.93), respectively^45^.

For qualitative analysis, microscopic histological observations were conducted, and specimens exhibiting histological changes were counted and recorded. The noted histological changes included chondrocyte death, fibrillation in the superficial and middle layers, fissuring, and matrix loss within the articular cartilage, as well as expansion, sclerosis, and osteocyte death in the subchondral bone.

### Histomorphometric analyses for articular cartilage

As described in previous protocols^18,41,48^, the assessment of cartilage thickness, matrix staining intensity with safranin O, and chondrocyte density within a 200-µm width from the center of the lesion were assessed using Adobe Photoshop CC imaging software (Adobe Systems, Inc., San Jose, CA, USA). The average of the cartilage thickness was calculated. Matrix staining intensity was quantified by calculating the average relative intensity of safranin O staining after grayscale conversion. For cell density analysis, the number of cell nuclei stained with hematoxylin was manually counted and divided by the area to derive the density.

### Immunohistochemical staining and analysis

Immunohistochemical staining was performed as previously described^48,49^. Paraffin sections were subjected to immunohistochemical staining with antibodies against Gremlin-1 (diluted 1:100, bs-1475R, BIOSS, Massachusetts, USA), PRG4 (diluted 1:3000, MABT401, MERCK, Tokyo, Japan), HYBID (diluted 1:100, 21129-1-AP, Proteintech, Tokyo, Japan), MMP 13 (diluted 1:50, 18165-1-AP, Proteintech, Tokyo, Japan), and ADAMTS 5 (diluted 1:50, ab41037, Abcam, Tokyo, Japan). For Gremlin-1, HYBID, MMP13, and ADAMTS5, using Adobe Photoshop CC imaging software (Adobe Inc., San Jose, USA), the positive cell density was calculated by manually counting the number of positive cells in the articular cartilage within a 200-μm width from the center of the lesion and then dividing by the area. For PRG4, after the conversion to grayscale, the relative staining intensity was calculated at a depth of 50 μm from the surface within the 200-μm wide center of the lesion.

### Sample size and statistical analysis

All statistical analyses were performed using the JMP 14 software (SAS Institute, Cary, NC, USA) in accordance with the instructions and guidance of the Specially Appointed Professor in the Division of Biostatistics (TT). On the basis of the expected histological changes in the cartilage at 14 days postoperatively in our previous study results^18,41^, we defined the time to evaluate the primary endpoint as 14 days postoperatively. Using our previous data^41^, we calculated the required sample size for the main parameters of the OARSI score, cartilage thickness, and chondrocyte density using G-power 3.1. (free; available at https://www.psychologie.hhu.de/arbeitsgruppen/allgemeine-psychologie-und-arbeitspsychologie/gpower.html)^50^. As a result, the sample sizes required for these parameters were calculated to be four, three, and six, respectively, with a power of 0.8 and a significance level of *p* < 0.05. Therefore, the experiment was conducted with a sample size of six.

Descriptive statistics were calculated, and the OARSI score is presented as the median with an interquartile range. Body weight and histomorphometric and immunohistochemical data were presented as means with standard deviations. The normality of all data was assessed using the Shapiro–Wilk test. To evaluate the differences between the CON group and each period within the OA group, analysis of variance was conducted using parametric continuous data. A post-hoc Dunnett’s test was then conducted. In case of a significant difference in the Shapiro–Wilk test, the Kruskal–Wallis test was employed, and a post-hoc Steel test was performed. A significance level of *p* < 0.05 was considered statistically significant for all the analyses. Precise *p*-values are provided in the figures and supplementary material.

### Supplementary Information

Supplementary Tables.

## Data Availability

All data generated or analyzed during this study are included in this published article and its Supplementary Information files.
