# Supplementary material for: Histological and immunohistochemical analyses of articular cartilage during onset and progression of pre- and early-stage osteoarthritis in a rodent model
Source: Sci Rep. 2024 May 8;14:10568. doi: 10.1038/s41598-024-61502-8 (PMC11079058; doi:10.1038/s41598-024-61502-8)
Supplement: Supplementary file 1 — Supplementary Tables. [file 41598_2024_61502_MOESM1_ESM.pdf]

S1 Table. The body weights of the animals during the experiment.

CON group
After 1 week of preliminary keeping
292.7 ± 10.3<br>(283.1–302.3)

OA group			
	At the start	At the end of the experiment	P value (95% CI)
At 1 day	294.3 ± 8.8<br>(285.0–303.5)	285.6 ± 8.9<br>(276.2–295.0)	0.1218<br>(-2.7–20.0)
At 3days	295.8 ± 13.0<br>(282.1–309.5)	301.1 ± 4.2<br>(296.7–305.5)	0.3777<br>(-8.3–19.0)
At 7 days	292.8 ± 12.6<br>(279.5–306.1)	298.5 ± 14.9<br>(282.8–314.1)	0.4941<br>(-12.1–23.4)
At 10 days	289.5 ± 12.4<br>(276.4–302.5)	303.8 ± 12.3<br>(290.8–316.7)	0.0733<br>(-1.6–30.2)
At 14days	295.0 ± 6.8<br>(287.7–302.2)	318.6 ± 10.7*<br>(307.4–329.9)	0.0011<br>(12.0–35.2)

Unit: gram, Mean ± SD (95% CI)

* There was a significant difference compared to the start of the experiment. $P < .05$ for all.

S2 Table. The OARSI score and histomorphometric results of articular cartilage.

The OARSI score

	CON group			OA group		
	Stage	Grade	Score	Stage	Grade	Score
At start	0.0 (0.0–0.0)	0.0 (0.0–0.0)	0.0 (0.0–0.0)	—	—	—
At 1 days	—	—	—	0.0 (0.0–0.0)	0.0 (0.0–0.0)	0.0 (0.0–0.0)
At 3 days	—	—	—	0.5 (0.0–1.0)	0.5 (0.0–1.0)	0.5 (0.0–1.0)
At 7 days	—	—	—	3.0* (2.0–3.0)	2.0* (1.7–2.0)	5.0* (3.7–6.0)
At 10 days	—	—	—	3.0* (3.0–4.0)	3.0* (2.0–3.0)	9.0* (7.5–9.7)
At 14 days	—	—	—	3.0* (3.0–4.0)	3.5* (3.0–4.0)	12.0* (9.0–13.0)

Median (lower quartile–upper quartile)

* There was a significant difference from the CON group. $P < .05$ for all.

**Grade**	P value	95% CI
At 1 days	1.0000	0.0–0.0
At 3 days	0.2467	0.0–1.0
At 7 days	0.0083*	1.0–2.0
At 10 days	0.0107*	3.0–4.0
At 14 days	0.0100*	2.0–3.0

**Stage**	P value	95% CI
At 1 days	1.0000	0.0–0.0
At 3 days	0.2467	0.0–1.0
At 7 days	0.0100*	2.0–3.0
At 10 days	0.0100*	3.0–4.0
At 14 days	0.0100*	3.0–4.0

**Score**	P value	95% CI
At 1 days	1.0000	0.0–0.0
At 3 days	0.2467	0.0–1.0
At 7 days	0.0113*	3.0–6.0
At 10 days	0.0113*	6.0–12.0
At 14 days	0.0115*	9.0–16.0

* There was a significant difference from the CON group. $P < .05$ for all.

Cartilage thickness

	CON group	OA group	P value (95% CI)
At start	0.278 ± 0.019 (0.258–0.299)	—	—
At 1 days	—	0.269 ± 0.019 (0.248–0.290)	0.9859 (-0.055–0.028)
At 3 days	—	0.259 ± 0.032 (0.225–0.292)	0.6312 (-0.065–0.045)
At 7 days	—	0.227 ± 0.026 (0.200–0.255)	0.0222* (-0.107–-0.020)
At 10 days	—	0.238 ± 0.015 (0.221–0.254)	0.0222* (-0.080–-0.018)
At 14 days	—	0.200 ± 0.042 (0.155–0.244)	0.0222* (-0.137–-0.026)

Unit: μm, Mean ± SD (95% CI)

* There was a significant difference from the CON group. *P* < .05 for all.

Matrix intensity

	CON group	OA group	P value (95% CI)
At start	145.0 ± 6.7 (137.9–152.1)	—	—
At 1 days	—	121.0 ± 7.2 (113.4–128.6)	0.0013*
At 3 days	—	117.5 ± 9.1 (107.9–127.2)	0.0003*
At 7 days	—	130.1 ± 15.4 (113.9–146.3)	0.0645
At 10 days	—	176.6 ± 10.1 (165.9–187.3)	< 0.0001*
At 14 days	—	170.9 ± 9.8 (160.6–181.2)	0.0006*

Unit: pixel value, Mean ± SD (95% CI)

* There was a significant difference from the CON group. *P* < .05 for all.

Chondrocyte density

	CON group	OA group	P value (95% CI)
At start	1023.1 ± 98.8 (919.4–1126.8)	—	—
At 1 days	—	1128.6 ± 64.0 (1061.4–1195.9)	0.5698
At 3 days	—	1054.3 ± 144.7 (902.3–1206.2)	0.9945
At 7 days	—	983.9 ± 194.0 (780.2–1187.5)	0.9846
At 10 days	—	422.9 ± 190.6 (222.7–623.0)	< 0.0001*
At 14 days	—	653.3 ± 100.2 (548.1–758.6)	0.0004*

Unit: cells/mm^2, Mean ± SD (95% CI)

* There was a significant difference from the CON group. $P < .05$ for all.

S3 Table. Immunohistochemical results

Positive cells density of Gremlin-1

	CON group	OA group	P value (95% CI)
At start	669.5 ± 74.4 (591.4–747.7)	—	—
At 1 days	—	911.4 ± 172.7 (730.1–1092.7)	0.0222* (28.2–383.7)
At 3 days	—	497.0 ± 189.1 (298.5–695.4)	0.3112 (-528.2–211.5)
At 7 days	—	425.2 ± 185.1 (230.9–619.5)	0.2316 (-530.4–205.6)
At 10 days	—	177.3 ± 37.8 (137.6–217.1)	0.0222* (-629.0–-325.0)
At 14 days	—	196.6 ± 94.9 (97.0–296.2)	0.0222* (-637.6–-169.2)

Unit: cells/mm^{2}µm, Mean ± SD (95% CI)

* There was a significant difference from the CON group. $P < .05$ for all.

Positive cells density of HYBID

	CON group	OA group	P value (95% CI)
At start	510.1 ± 91.6 (414.0–606.3)	—	—
At 1 days	—	812.9 ± 213.2 (589.0–1036.7)	0.0148*
At 3 days	—	627.7 ± 130.7 (490.4–764.9)	0.6160
At 7 days	—	475.8 ± 275.6 (186.5–765.1)	0.9959
At 10 days	—	205.1 ± 43.5 (159.3–250.8)	0.0139*
At 14 days	—	242.3 ± 122.1 (114.2–370.5)	0.0355*

Unit: cells/mm^{2}μm, Mean ± SD (95% CI)

* There was a significant difference from the CON group. $P < .05$ for all.

Intensity of PRG4

	CON group	OA group	P value (95% CI)
At start	161.5 ± 3.4 (157.8–165.2)	—	—
At 1 days	—	171.9 ± 9.7 (161.7–182.1)	0.2302 (-14.9–21.3)
At 3 days	—	161.0 ± 12.2 (148.1–173.9)	0.9938 (-25.8–17.2)
At 7 days	—	122.6 ± 22.6 (98.8–146.3)	0.0219* (-66.1–-34.3)
At 10 days	—	111.1 ± 8.0 (102.7–119.6)	0.0219* (-64.1–-34.3)
At 14 days	—	143.4 ± 15.8 (126.8–160.1)	0.1664 (-48.5–4.0)

Unit: pixel value, Mean ± SD (95% CI)

* There was a significant difference from the CON group. *P* < .05 for all.

Positive cells density of MMP13

	CON group	OA group	P value (95% CI)
At start	650.7 ± 75.5 (571.4–730.1)	—	—
At 1 days	—	895.8 ± 179.7 (707.1–1084.5)	0.0027*
At 3 days	—	581.2 ± 136.6 (437.8–724.5)	0.7156
At 7 days	—	382.8 ± 66.4 (313.0–452.6)	0.0010*
At 10 days	—	197.9 ± 67.9 (126.6–269.2)	< 0.0001*
At 14 days	—	187.1 ± 88.1 (94.6–279.5)	< 0.0001*

Unit: cells/mm^{2}μm, Mean ± SD (95% CI)

* There was a significant difference from the CON group. *P* < .05 for all.

Positive cells density of ADAMTS5

	CON group	OA group	P value (95% CI)
At start	471.7 ± 26.6 (443.8–499.7)	—	—
At 1 days	—	599.3 ± 77.7 (517.7–680.9)	0.1181 (-62.2–206.2)
At 3 days	—	314.3 ± 58.1 (253.3–375.2)	0.0222* (-254.1–-24.7)
At 7 days	—	343.8 ± 201.7 (132.1–555.6)	0.8482 (-375.3–197.0)
At 10 days	—	108.4 ± 23.3 (84.0–132.9)	0.0222* (-434.9–-309.0)
At 14 days	—	154.1 ± 84.7 (65.1–243.1)	0.0222* (-254.1–-24.7)

Unit: cells/mm^{2}μm, Mean ± SD (95% CI)

* There was a significant difference from the CON group. *P* < .05 for all.
